# Redefining Arctic boundaries in a changing climate: interdisciplinary perspectives on governance strategies

**DOI:** 10.1080/1088937X.2024.2359926

**Published:** 2024-06-13

**Authors:** Ugo Nanni, Patricia DeRepentigny, Aapo Lundén, Virginija Popovaitė, Yiyi Shen, Ilker K. Basaran, Natália Duarte Neubern, Llucia Mascorda-Cabre, Alec Bennett, Tiril Vold Hansen, Felicity A. Holmes, Eleni Kavvatha, Alexandra Meyer, Abhay Prakash, Aleksandra Wołoszyn

**Affiliations:** a Department of Geosciences, University of Oslo, Oslo, Norway; b Climate and Global Dynamics Laboratory, National Center for Atmospheric Research, Boulder, CO, USA; c Geography Research Unit, University of Oulu, Oulu, Finland; d Faculty of Social Sciences, Nord University, Bodø, Norway; e Department of European, International and Comparative Law, University of Vienna, Vienna, Austria; f Department of Maritime Studies, Texas A&M University, Galveston, TX, USA; g International Relations Institute, Pontifical Catholic University of Rio de Janeiro, Rio de Janeiro, Brazil; h School of Biological and Marine Sciences, University of Plymouth, Plymouth, United Kingdom; i College of Business and Security Management, University of Alaska Fairbanks, Fairbanks, AK, USA; j Department of Physical Geography, Stockholm University, Stockholm, Sweden; k Centre de recherche en science politique, Institut d'Études Européennes, UCLouvain Saint-Louis, Brussels, Belgium; l Department of Social and Cultural Anthropology, University of Vienna, Vienna, Austria; m Institute of Geography and Regional Development, University of Wrocław, Wrocław, Poland

**Keywords:** Arctic, environmental change, boundaries, Svalbard, risk perception, multi-scale analysis

## Abstract

The Arctic rapidly transforms due to global warming and increased human activities, triggering complex changes at unprecedented speeds that challenge conventional institutional responses. We analyse these changes through the lenses of social, political, and environmental boundaries and investigate their impacts on both inhabitants' livelihoods and the region's political framework. Employing an interdisciplinary approach, we highlight the complexities of understanding the interplay among global, regional, and local dynamics in an era where human and non-human aspects are entwined. Our analysis concentrates on three areas: definition of the Arctic; legal disputes concerning the waters around the Svalbard Archipelago; evolving natural hazards and societal risk perceptions in Longyearbyen. Through these examples, we underscore the intricate nature of social, political, and ecological changes and how they challenge current boundary-making processes. By combining knowledge from different systems and scales, our research reveals the interplay between policy-driven science, science-influenced policy, and performative behaviors in reshaping today's Arctic borders and boundaries. We particularly emphasize how climate change is challenging borders and advocate for a departure from static definitions, towards the formulation of environmentally conscious, socially just, and politically viable policies, acknowledging the new biophysical realities of the Anthropocene.

## 1. Introduction

The Arctic has long been considered a barrier region due to its limited access and communication, similar to other remote areas such as the Amazon, the Himalayas, Siberia, or the Sahara desert (Sörlin, 2017, p. 272). Often characterized as cold, remote, and isolated, it is home to almost four million people, including various indigenous groups, and is of major ecological, geopolitical, military, and economic importance (Coumou et al., 2018; Henderson et al., 2021; Nowlan, 2001). The rapid pace of modern environmental changes transcends the conventional conception and function of established boundaries, from social to physical and local to global boundaries (Kuus, 2020; Lysaker, 2023; Milkoreit, 2023; Rockström et al., 2023). In the Arctic region, the impacts of climate change are both pronounced and symbolic of broader geopolitical and ecological shifts (Martin & Cometti, 2021). Consequently, change is becoming the norm in the Arctic, with environmental shifts intensifying in number and magnitude (Rantanen et al., 2022, e.g. atmospheric warming is nearly four times higher than the rest of the globe), affecting areas within and beyond its borders (AMAP, 2021; Hinzman et al., 2005; Kim et al., 2023; Meredith et al., 2019; Post et al., 2009; Serreze, 2018). Change also happens at the level of lived and experienced spaces and imaginaries^1^ as those living in the Arctic are faced with growing risks and forced to adapt their infrastructures and traditional livelihoods to the growing uncertainty of the state of the environment they depend on (Ford et al., 2019, 2006). In response to such changes, our conception of the Arctic is gradually being reshaped, yet only a limited number of studies have explored the impact of climate change on the boundaries within and across the Arctic (Dalby, 2021).

Today's Arctic region is subject to two contrasting perspectives. Geographically, the political boundaries of the Arctic and its transnational institutions and coalitions (e.g. the Arctic Council, the Arctic Five, Inuit Circumpolar Council, the Sámi Council) offer opportunities for cooperation among stakeholders (Minghi, 1963). These organizations serve as platforms for dialogue that includes governments, Indigenous peoples, scientific organizations, and civil society. This perspective is manifested, for instance, in the narrative of seeing the Arctic as a region of peace and cooperation (Young, 2011), and supported by scholars describing the Arctic as a Polar Mediterranean (Steinberg et al., 2015). On the other hand, from a perspective of state territoriality, sovereignty, and economic opportunities, the Arctic is seen as a frontier-like space of untapped potential and exploration, leading to competition and tensions among states (Kristoffersen & Langhelle, 2017; Newman & Paasi, 1998). For instance, the United Nations Convention on the Law of the Sea (UNCLOS, 1982) lets coastal states claim sovereignty and extend the 200-nautical miles continental shelf limit (Carlson et al., 2013, Figure 2), nationalizing and internationalizing the ocean and creating a bundle of rights (Byers, 2010; Waller, 2018). However, changing bio-physical systems in the Arctic have triggered the process of de-bordering in the field of maritime legislation (Mezzadra & Neilson, 2013). The Arctic has thus become one of the most speculated regions in the world, whether for its strategic and economic potential (National Research Council, 2014) or its deteriorating natural environment (Bennett, 2020). This dynamics highlights the temporal nature of boundaries and the human impulse to establish new ones. While transnational institutions can promote cooperation among stakeholders, the emphasis on state territoriality, sovereignty, and economic boundaries may result in competition and tension. In response to the shifting climatic paradigm, the Arctic region also emerge as a potential cornerstone in the reconfiguration of human habitats (Vince, 2022). Perceiving the Arctic as a global resource to battle climate change (e.g. rare-earth minerals), an opportunity for prosperity (e.g. oil and gas), or as the homeland of people (Kristoffersen, 2014; Kristoffersen & Langhelle, 2017) has significant implications for how and whose boundaries matter. As a result, the perspective(s) on changing Arctic boundaries may be optimistic, opportunistic, or grim.

In this paper, we investigate the changing Arctic through the lens of shifting social, political, and environmental boundaries (Coote, 2023; Kebir et al., 2023; Nuttall, 2023). Particularly, we examine how trans-boundary changes intersect and interact with historically-based static borders in the Arctic region, affecting both the lives of its inhabitants and the political landscape (Carter et al., 2021; Kuus, 2020). We focus on boundaries as ‘zones of transition’ between diverse societies, environments and power centers (Anderson & O'dowd, 1999), rather than solely in term of legal demarcations (Prescott, 2014), and investigate their evolution as symptoms and manifestations of changes. By employing a multi-scalar interdisciplinary perspective, we highlight the challenges of understanding the convolution of global, regional, and local changes in the Arctic at a time where the human and non-human dimensions are inseparably linked (Folke et al., 2021; Stephenson, 2018). First, we discuss how the evolution of natural boundaries, historically used to define the Arctic, challenges our perception of the Arctic as a region (Section 3). Second, we investigate how legal rights over the waters surrounding the Svalbard Archipelago are disputed and shaped by evolving bio-physical boundaries (Section 4). Finally, we explore how changing climatic conditions challenge risk management by analyzing the evolution of risk boundaries in the town of Longyearbyen in the Norwegian Arctic (Section 5). Through these three examples, we draw attention to the complexity of social, political, and ecological changes and how they challenge current boundary-making processes in the Arctic region. By combining knowledge from different systems and at different scales, our study reveals the interplay between policy-based science, science-based policy and performative behaviour behind the complex dynamics of Arctic borders and boundary-making.

## 2. Theoretical framework on boundaries

A fundamental insight into boundaries lies in their intricate and hybrid nature, weaving together natural, political, and societal processes stemming from changes across various scales (Pic, 2022). To analyze the emerging and complex interactions in the Arctic, our study focuses on changing boundaries as symptoms (Minghi, 1963) of the current human-induced climate crisis. According to Newman and Paasi (1998), human-made boundaries are contextual and operate across multiple scales. On the global scale, these boundaries are often geopolitical and grounded in economic landscapes; at the state level, the focus may revolve around ongoing nation-building and national identity; and at the local scale, boundaries are both tangible and perceived, falling within the realm of everyday life experiences. Building upon this categorization, we focus on how different boundaries in the Arctic operate across different scales and become increasingly porous due to ongoing environmental changes, which puts their relevance into question.

Defining boundaries with political and practical relevance necessitates regional as well as global knowledge production. Science-based policy and policy-based science have emerged as two interconnected concepts playing a pivotal role in shaping boundaries (Strassheim & Kettunen, 2014). Science-based policy refers to the formulation and implementation of policies grounded in scientific evidence and understanding. For example, Arctic cooperation and knowledge regarding the bio-accumulation of persistent organic pollutants (Rottem, 2017) or the Agreement on the Conservation of Polar Bears (1973) showcase policies driven rather directly and efficiently from scientific endeavors (Brower et al., 2002). On the other hand, policy-based science is motivated or prioritized based on other goals by policy agendas or the need to address specific policy areas. This could lead to selective knowledge in order to fit pre-existing beliefs or influence power relations. For instance, the prioritization of oil and gas exploration in the Arctic (Gautier et al., 2009), driven by considerations of energy security, geopolitics, and economic interests, exemplifies how policy objectives intertwine political and environmental realms in the region. Consequently, it raises the question of whether responses to environmental disruptions today rely on scientific knowledge only or also on the ability of science to translate into politically relevant impacts (Lövbrand et al., 2015), highlighting the performative nature of knowledge production and its interplay with politics.

Knowledge production and its impact on boundary-making in today's Arctic not only guides but constrains what and how it is governed. The very process of defining the Arctic as a unit serves as a source of legitimacy and relevance for its operationalization, portraying the Arctic region as a boundary object. In other words, it is a region resilient enough to ‘*adapt to local needs and constraints of the several parties employing them, yet robust enough to maintain a common identity across sites*’ thus providing ‘*different meanings in different social worlds*’ (Bowker & Star, 1999, p. 297). This complexity underlines the necessity to move beyond a linear model of expertise (Beck, 2011), especially in the context of environmental changes outpacing the ability of political institutions to respond to it (Rockström et al., 2023). By integrating examples of Arctic change from different scales and epistemologies and exploring their implications on boundary-making in the region, we pave the way for a more resilient Arctic.

## 3. The many Arctics of the Anthropocene

In this section, we examine the demarcation process that resulted in the first internationally-shared operational definition of the Arctic. We then explore how ongoing changes in both the natural and human environments are challenging the suitability of this definition (Stephenson, 2018).

### 3.1. Defining the Arctic

Defining the Arctic is an essential step for investigating the socio-environmental dynamics at stake under current climate change. However, in contrast to the Antarctic which is defined fairly directly based on the land surrounding the South Pole (Nowlan, 2001), no omnipotent definition of the Arctic or even its involved states exists, and rightly so. Conceptually, the Arctic refers to multiple constellations and scales (e.g. high Arctic, low Arctic, sub-Arctic; Hamelin, 1968), ranging from natural, objective, and calculable definitions (e.g. phytogeographic boundaries such as the tree line or the presence of permafrost, climatic boundaries based on average temperatures or the extent of sea ice; Linell & Tedrow, 1981; Stonehouse, 1989; Woo & Gregor, 1992), to places and latitudes (e.g. north of the Arctic Circle at about 66°33′N; O'Rourke, 2011), to imaginaries and socially constructed ideas of Arctic environments and cultures (Lehtinen, 2019; Saarinen & Varnajot, 2019; Tennberg, 2022). It is only after World War II, with the threat of the cold war and the generation of systemic knowledge about the Arctic's role in the climate system, that the Arctic became the global indicator of contemporary change (Sörlin, 2017).

**Figure 1.**
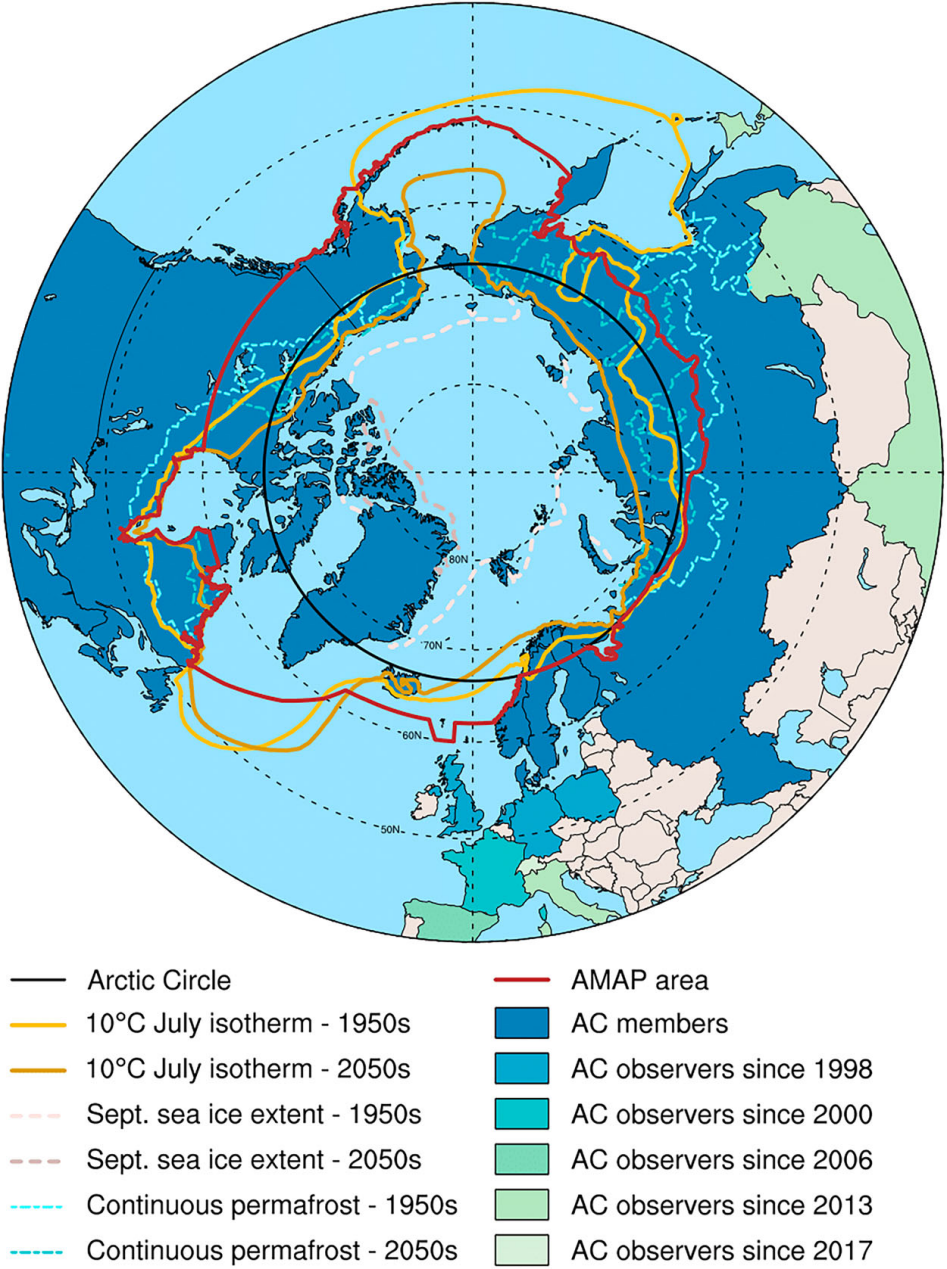
Different definitions of the Arctic based on geographical, physical, and political characteristics and their evolution with time. The boundary of the Arctic Monitoring and Assessment Programme assessment area (red solid line) was defined based on a compromise among various definitions and incorporates elements of the Arctic Circle (black solid line), political boundaries, vegetation boundaries, permafrost limits, and major oceanographic features (AMAP, 1998). The northward shrinkage of natural boundaries over time is illustrated by the change in September Arctic sea ice extent, 10℃ July isotherm and continuous permafrost line over different decades using climate simulations from the CESM2-LE (see Appendix for more details). The southward diffusion of geopolitical interests in the Arctic is shown by the different shading of Arctic Council (AC) members and observer states.

In 1991, the Arctic Monitoring and Assessment Programme (AMAP) was established to implement components of the Arctic Environmental Protection Strategy as adopted at the time by the Ministers of Environment of the eight Arctic Countries (Canada, Denmark, Finland, Iceland, Norway, Sweden, the Russian Federation, and the United States). As one of the working groups of the Arctic Council, AMAP was given the mandate to monitor and assess the pollution of the Arctic environment and to provide policy recommendations based on scientific assessments (AMAP, 1998, Chapter 2). To do so, AMAP needed to define a region of focus for its assessment activities that went beyond the simplistic definition of the Arctic as the land and sea area north of the Arctic Circle. This boundary-making process was necessary to shape a region suitable to address global connections associated with long-range transport of contaminants. Given the many definitions of the Arctic based on physical-geographical characteristics (e.g.10℃ July isotherm, treeline, extent of continuous permafrost, oceanic currents) and those based on political and administrative considerations, the boundary-making process turned out to be a complex endeavor (Dalby, 2021; Scoles, 2018). In the end, the borders drawn by AMAP were based on a compromise among various knowledge production systems, encompassing the terrestrial and marine areas north of the Arctic Circle, north of 62°N in Asia and 60°N in North America, and including the marine areas north of the Aleutian chain, Hudson Bay, and parts of the North Atlantic Ocean including the Labrador Sea (Figure 1, red line).

AMAP was the first attempt at defining the Arctic based on a global and interdisciplinary perspective, effectively regionalizing environmental concerns within the context of global systems that are intimately connected. It has been highly successful in identifying emerging issues, framing them for consideration in policy venues, and raising their visibility on the policy agenda (Kankaanpää & Young, 2012). Particularly, the scientific assessments provided by AMAP have contributed, notably, to reduce pollution not only in the Arctic but at the planetary level, especially the contaminant exposure of indigenous and local communities with a traditional lifestyle (Platjouw et al., 2018; Reiersen et al., 2024). This initiative allowed for systematization, expansion, and coordination of Arctic research and monitoring efforts. In addition, coordination through AMAP is acting as a significant peace process to keep the Arctic as a low-tension area where countries have eased political questions through dialogue and joint work (Reiersen et al., 2024; Rødven & Wilson, 2022).

### 3.2. The double bind of Arctic change

Since the establishment of AMAP in 1991, the effects of climate change on the Arctic have become increasingly apparent (Serreze, 2018) and are happening faster than at global scale (i.e. the Arctic is warming four times faster Rantanen et al., 2022). This amplified warming has led to continued loss of sea ice in all seasons (Kim et al., 2023; Stroeve & Notz, 2018), ice losses from glaciers and ice caps (Ding et al., 2023), thawing of permafrost (Hjort et al., 2018, 2022), changes in the oceans such as the Atlantification of the Arctic (i.e. increased influence of Atlantic water in the Arctic Ocean because of reduced ice cover and inflow of warm Atlantic water; Årthun et al., 2012). Future projections suggest a continued bio-physical shrinking of the Arctic. As a result, the climatic boundaries previously used during the boundary-making process of the Arctic (i.e. 10°C July isotherm, minimum sea ice coverage, extent of continuous permafrost) are now shaping a shrunken Arctic retracting north compared to in the past (Figure 1).

Simultaneously, the Arctic is expanding southward in the political and cultural sphere. In tourism, the Arctification process refers to the spread of the Arctic brand to more southerly destinations (Bohn & Varnajot, 2021; Marjavaara et al., 2022) and has been identified as a contributing factor to tourism growth in the North (Lundmark et al., 2020; D. K. Müller & Viken, 2017; Palma et al., 2019; Varnajot & Saarinen, 2021). One example of this is the use of Arctic-themed branding and marketing materials, such as images of polar bears, icebergs, and snow-covered landscapes, to promote destinations that are located far above the Arctic Circle. In terms of international relations and transnational governance, Arctic politics have undergone significant expansion and transformation in recent decades as the region gained increasing geopolitical importance due to its vast natural resources as well as its strategic location as a global shipping route (Evengård et al., 2015). As such, the Arctic Council has expanded its membership from the original eight member countries to include observer countries from around the world, including China, South Korea, India, and Singapore (Figure 1, color-shaded countries; Nord, 2015). The Council has also placed greater emphasis on the role of indigenous peoples in the Arctic, recognizing their unique perspectives, knowledge, and rights to self-determination (Koivurova, 2010). Thus, the Arctic has expanded politically and issue-wise over the years in response to the growing geopolitical importance of the region, ongoing environmental changes, and the increasing international attention it receives.

### 3.3. Towards a hybrid Arctic?

Given the transformative changes occurring in the Arctic, as described above, questions have arisen regarding the prospects for the Arctic Council and its effectiveness in the foreseeable future, especially considering the growing political tension between the Arctic countries and non-Arctic countries (Kankaanpää & Young, 2012). The Arctic's critical environmental significance poses a challenge to conventional territoriality and state-centric paradigms within the international dialogue and thus prompts a re-evaluation of regional identity, autonomy, and governance. This situation raises questions about who has the authority to decide the significance and goals of borders, and how these decisions are influenced by established maritime and terrestrial governance frameworks (Huebert, 2018; Lövbrand et al., 2015). As a result, the Arctic emerges as an increasingly renegotiated space, with constant de- and re-bordering dynamics. The interplay between the two trends described above – the shrinkage of the Arctic's physical boundaries and the expansion of its political relevance – highlights the need for a new approach to governance in the region. Indeed, as the Arctic becomes increasingly accessible and its boundaries are challenged by biophysical changes, there is a growing awareness that the traditional nation-state-centric approach to defining the region's borders is no longer sufficient (Kikkert & Lackenbauer, 2019; Stephenson, 2018).

## 4. Svalbard's evolving maritime zones

The Svalbard Archipelago, situated in the Norwegian Arctic, stands out due to its unique political and socio-economic history, as well as its central location in the Arctic. In this section, we examine the political challenges arising from the dispute over the maritime zone around Svalbard and how changes in the region's bio-physical boundaries are further impacting the legal and political borders of the region.

### 4.1. Disputes over legal boundaries

**Figure 2.**
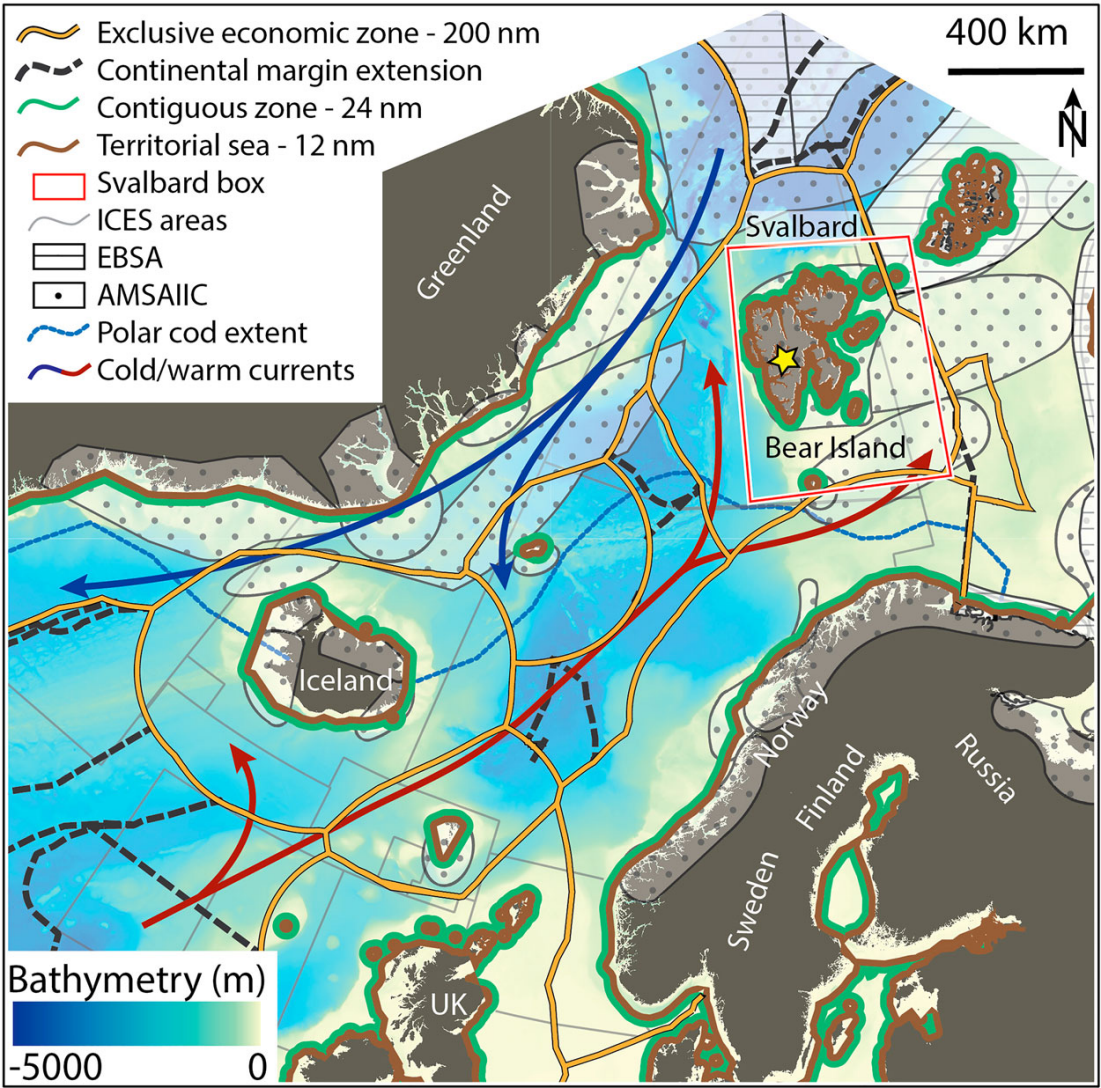
Map of the Svalbard Archipelago and its surroundings. The map displays the bathymetry of the region and the natural and legal boundaries surrounding the Svalbard box (red box). Maritime boundaries include territorial seas (12 nm from the shore, green lines), contiguous zone (24 nm from the shore, brown lines), exclusive economic zones (EEZs; 200 nm from the shore, yellow lines), extensions of the continental shelf from the exclusive economic zones (dashed black lines). Regulated areas include Arctic Ecological and Biological Significance Areas (EBSAs, dashed areas), Arctic marine areas of heightened ecological significance (AMSAIIC, dotted area) and areas from the International Council for the Exploration of the Sea (ICES, thin gray lines). Natural boundaries include the southern extent of the Polar Cod habitat (dashed blue line), the major warm oceanic currents (North Atlantic and Azores, Norwegian, West Spitsbergen, red arrows) that bring warm and saline Atlantic Water to high latitudes and the major cold oceanic currents (Transpolar Drift, East Spitsbergen and East Greenland, blue arrows) that bring cold Arctic Water to low latitudes. Land masses are shown in dark gray. The yellow star shows the location of Longyearbyen in Figure 4.

Since 1925, the Svalbard Treaty dictates the legal status of the Svalbard Archipelago (Basaran, 2023; Jensen, 2020). The Treaty, ratified by 46 parties, grants Norway complete sovereignty over the ‘Svalbard box’, described as covering ‘the Archipelago of Spitsbergen, comprising, with Bear Island or Beeren-Eiland, all the islands situated between 10° and 35° longitude East of Greenwich and between 74° and 81° latitude North’ (The Svalbard Treaty, 1920, Article 1; Red box in Figure 2). This defined area serves as a legal boundary, outlining the region where signatory states have equal access and economic rights (e.g. fishing, hunting, cruising; The Svalbard Treaty, 1920, Article 2).

In 1982, the United Nations Convention on the Law of the Sea (UNCLOS, 1982) was established as the most comprehensive framework for managing marine natural resources of the world's oceans and seas. UNCLOS confers jurisdictional rights on coastal states through various zonal delimitations, including a territorial sea (12 nautical miles - nm, Article 3), a contiguous zone (24 nm, Article 33), an exclusive economic zone (EEZ; 200 nm, Article 57) extending from the territorial sea, and an extension of the EEZ on the continental shelf (Article 76; Figure 2). Notably, the EEZ grants the coastal state economic and resource-related rights, allowing it to explore, exploit, conserve, and manage natural resources within its designated EEZ.

These two international agreements establish different legal and political boundaries, leading to disputes regarding jurisdiction over Svalbard waters. The central question revolves around whether it is the Svalbard Treaty's principles of equal access and non-discrimination, or the coastal state's sovereign rights under UNCLOS that should rule the Svalbard maritime area (Pedersen, 2006, 2020). The Svalbard Treaty explicitly mentions ‘territorial waters’ as the area of application. However, when the Treaty was ratified, modern concepts including the continental shelf, EEZ, and Fishery Protection Zone (Hønneland, 2001) had not yet been developed. The term ‘territorial waters’ only provided states with an approximate distance of 3–4 nm from their shore, compared to the 12 nm of territorial sea defined by UNCLOS 57 years later (Figure 2). Norway claims, since 1970, that interpreting the Svalbard Treaty accurately confines it to Svalbard's territorial waters (i.e. 4 nm from the shore Dyndal, 2014, p. 83), prohibiting other signatory states from extending their rights beyond this area and benefiting only Norway in conducting its economic activities in the region. Other signatory states, particularly Russia and the European Union, argue that the Treaty should be interpreted according to the current maritime delimitation principles under UNCLOS, allowing for an equal distribution of resources over the Svalbard's EEZ (i.e. 200 nm from the shore Pedersen, 2006, p. 1). These different interpretations rest upon the nature of Svalbard's continental shelf (i.e. the stretch of the seabed adjacent to the shore). If Svalbard has its own continental shelf and consequently, under UNCLOS, it's own EEZ, the Svalbard Treaty's non-discriminatory exploration rights for all signatory states would apply to that broader area (i.e. 200 nm from the shore compared to 4 nm from the shore). Conversely, if Svalbard's continental shelf is a contiguous extension of the Norwegian mainland seabed, Norway would have sovereign and exclusive rights over these areas independently of the Svalbard Treaty.

### 4.2. Human and non-human boundary interactions

**Figure 3.**
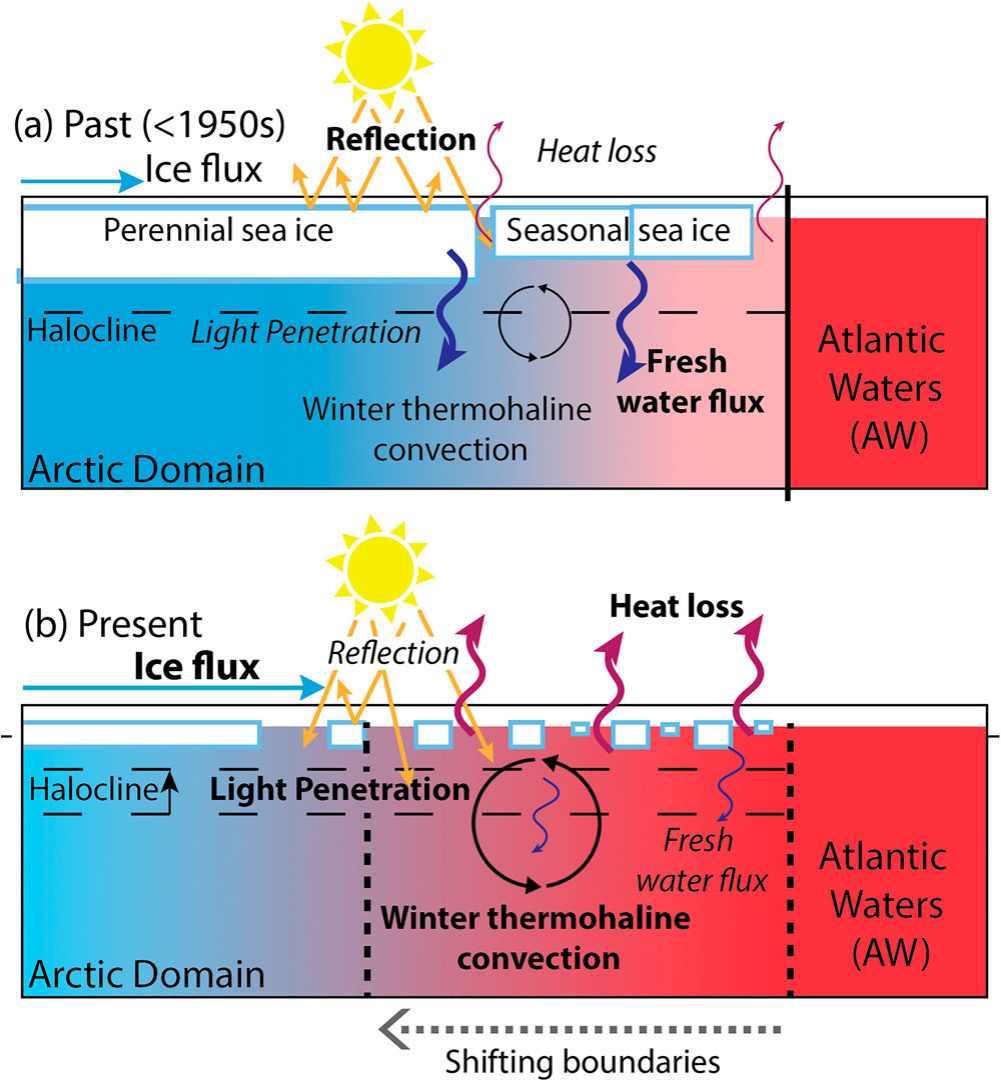
Shifting physical boundaries between the Atlantic and Arctic Oceans. Illustration of the different processes governing the ocean heat budget at the interface of the Atlantic (red) and Arctic (blue) Oceans, with the size of the arrows indicating the strength of the corresponding mechanism. (a) Past climate (pre-1950s): presence of a perennial multi-year sea ice pack provided an effective barrier between the ocean and atmosphere. Winter heat loss was negligible, thus limiting ocean circulation-driven differences in temperature and salinity. Furthermore, shortwave heating during summer generated substantial freshwater flux, which strengthened the ocean stratification. As such, a deeper halocline helped maintain the divide between the deep and the upper layer of the ocean. (b) Present climate: warming-induced loss of sea ice cover (Stroeve & Notz, 2018) drives substantially higher (winter) ocean heat loss and stronger thermohaline convection (Polyakov et al., 2010). Summer freshwater flux is also significantly limited. Thus, the barrier between the deep and upper ocean is diminished, warm Atlantic Waters at depth are able to drive more basal melt of sea ice (Polyakov et al., 2017), and the once distinct divide between the Atlantic and Arctic Oceans is quickly disappearing (a phenomenon referred to as Arctic Atlantification; Ingvaldsen et al., 2021). Reduced sea ice cover also lowers the surface reflectivity (albedo), causing more light and heat absorption by the ocean and increasing primary productivity (Bienhold et al., 2022; Hunt et al., 2016).

Concomitantly to the conflicting legal boundaries, the ocean surrounding Svalbard is experiencing significant changes due to human-induced climate change (Section 3). As a consequence, the region's bio-physical boundaries are progressively shifting (Figure 3), epitomised by a near halving of the end-of-summer Arctic sea ice cover since 1979 (Stroeve & Notz, 2018). The loss of the Arctic sea ice cover and the warming of the ocean have resulted in a weakening of the barrier – the halocline – between deeper warm waters and colder surface waters (Polyakov et al., 2010), leading to more sea ice basal melt and thus further weakening of the halocline (Polyakov et al., 2017). On one hand, the reduction of sea ice cover has caused increased light availability for primary productivity both in terms of magnitude and extent (Bienhold et al., 2022; Nicolaus et al., 2012, Figure 3). On the other hand, the weakening of the halocline has led to the northward advection of Atlantic Water, allowing the penetration of warmer water from the Atlantic ocean (i.e.Atlantification, Figure 3) and favouring enhanced nutrient availability (Tremblay et al., 2015). As a result, the Arctic ocean primary productivity has increased by 57% since 1998 (Bienhold et al., 2022), thus disrupting its ecological health (Bienhold et al., 2022; Hunt et al., 2016). Altogether, these transformations have led to a northward shift of marine organisms and ultimately, changes in commercially important fish stocks (i.e. borealization of of the Arctic), which brings into sharp focus the intricate interaction between shifting natural boundaries and legal-political interests with great socioeconomic implications in the region (von Biela et al., 2023).

Through the lens of changing and interacting human and non-human boundaries, we see that the challenges posed by the Svalbard maritime zone do not only impact Norway's sovereignty in a constitutive sense (i.e. the relationship between domestic and international boundaries) but also influences its functional sovereignty (i.e. the relationship between economic, political, and non-state boundaries). This duality illustrates how legal-political boundaries may need to adapt to changing natural boundaries. For instance, the debate over UNCLOS and the Svalbard Treaty, in conjunction with increasing environmental changes, poses significant challenges for fishery management organizations (e.g. the International Council for the Exploration of the Sea) and have already led to international conflicts over resource allocation (Hollowed et al., 2013; Mueter, 2022). This was recently illustrated by the Norwegian Supreme Court ruling on the validity of denying a foreign fishing company a license to catch snow crab on the continental shelf of Svalbard (Norwegian Supreme Court, 2023). The court unanimously decided that the equality rule in Articles 2 and 3 of the Svalbard Treaty applies to Svalbard's internal waters and maritime territory (12 nm) but not on the continental shelf of Svalbard. This decision aligns with Norway's longs tanding position. If the company had won the right to catch snow crab on the continental shelf, it would have had wider implications for access to natural resources including oil and gas, as snow crab is considered a sedentary species under UNCLOS.^2^

As atmospheric and ocean warming will continue and increase in the future, climate model projections indicate that the Arctic Ocean will likely become ice free during the summer season as early as the 2030s–2050s (Figure 1; Kim et al., 2023; Notz & SIMIP Community, 2020). As a result, current fisheries management and conservation zones, such as the Svalbard Fishery Protection Zone, the Ecological and Biological Significance Areas (Calado et al., 2009), and the Arctic marine areas of heightened ecological significance (Monitoring & Group, 2013), might become obsolete if the species they intend to protect are no longer present or their natural distribution has shifted (Figure 2). Indeed, certain species^3^ have already been identified as potential candidates for expansion into the high Arctic. The ongoing changes in natural boundaries have thus intensified a situation where political decisions are closely linked with industrial interests. Addressing these issues requires cooperation between the scientific community and marine managers at international and national levels to develop new monitoring strategies and ensure conflict resolution, sustainable stock management, and marine conservation (Galappaththi et al., 2022; Hollowed et al., 2013; Mueter, 2022).

## 5. Responses to evolving natural hazards in Longyearbyen

In this section, we focus on the interaction between environmental change and the resilience capacity of a local community with the example of changing avalanche risk in Longyearbyen, Svalbard. Specifically, we examine the town's response to two unprecedented snow avalanche events through the lens of changing perception of local risk boundaries.

We follow the definition of risk of Beck (2009) as being an anticipation of the unknown based on knowledge, but emphasizes that the definition itself is a product of negotiations. Within the context of the changing Arctic, we particularly focus on how ‘*global risks permeate and revolutionize everyday lifeworlds*’ (Beck, 2009). We investigate emerging risks faced by Arctic communities, as well as how they are defined through interlinked physical, social, and technical processes. While natural hazards themselves contribute to risk, exposure to that hazard (spatially and/or temporally) and vulnerabilities that contribute to impacts (e.g. low quality of construction, policy gaps, socio-economic status, etc.) act as modifiers of that risk, and often involve socially-driven components (UNDRR, 2020). Understanding a particular risk in isolation may understate the impact of compound or cascading risks (De Angeli et al., 2022; Fernández-Giménez et al., 2012). Reducing risk depends on understanding the complexity of the system, which includes both the physical aspects as well as perceived tolerance or acceptance of risk, and not least individual or cultural perceptions (Alessa et al., 2008; Renn et al., 2016). The boundaries on a risk map are therefore the result of complex interactions among these factors and among different knowledge systems. In this section, we take these boundaries and their fluctuation as a starting point to understand the challenge of living in a changing environment.

### 5.1. Longyearbyen, the gateway to the Arctic

**Figure 4.**
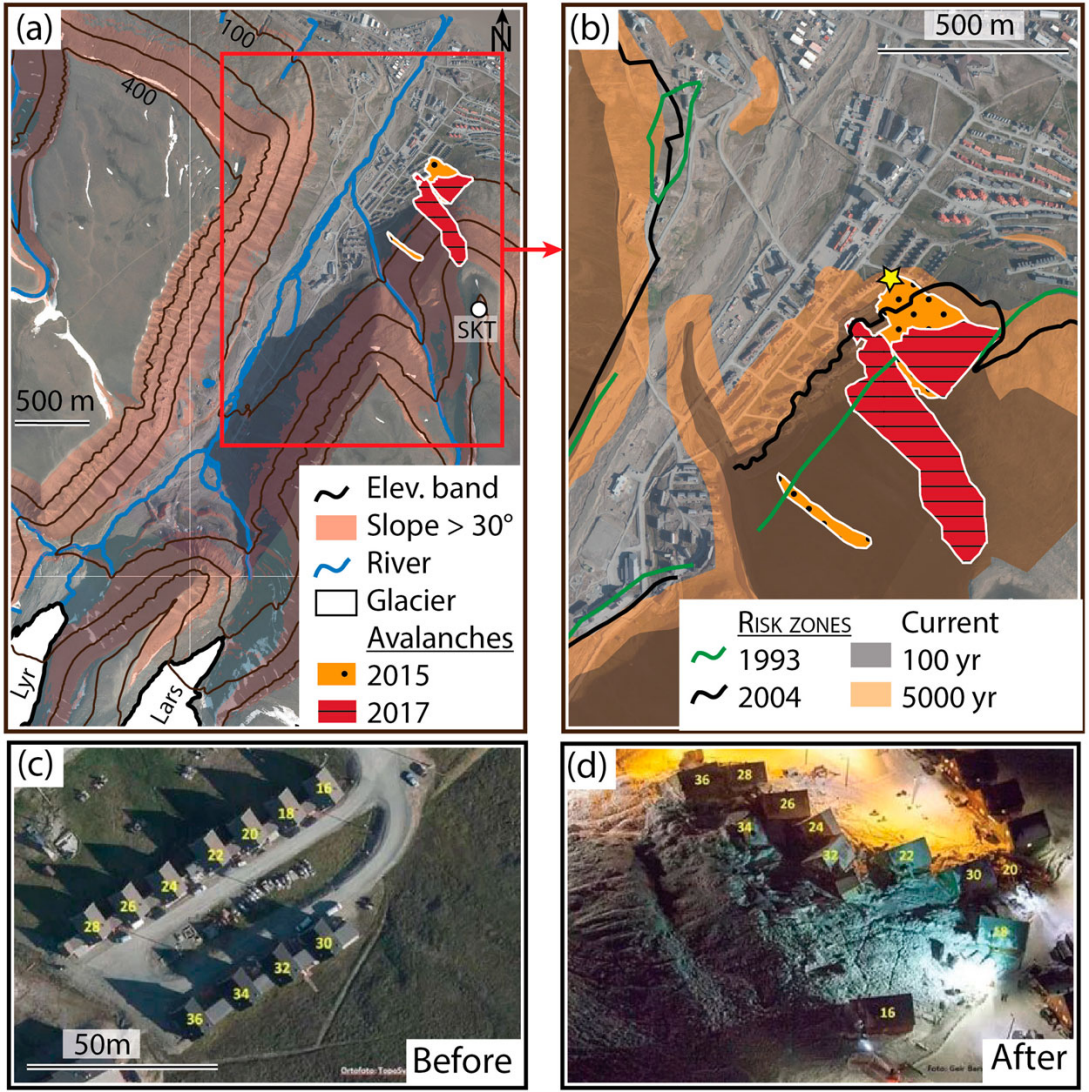
Topographic features and risk maps in Longyearbyen. (a) Satellite view of the city with its surrounding glaciers Longyearbreen (Lyr) and Larsbreen (Lars) from which the Longyearelva River originates (blue lines) and the surrounding mountains such as Sukkertopen (SKT) where avalanches initiated in 2015 (dotted orange patch) and 2017 (dashed red patch). Black lines indicate different elevation bands (in m) and shaded brown areas highlight slopes higher than 30°. (b) A zoomed-in view of the Lia neighborhood, which was impacted by the two avalanches, along with overlying risk boundaries. Green lines indicate risk boundaries drawn in 1993, black lines indicate those drawn in 2004, and black and orange shaded areas show risk areas drawn after the 2015 avalanche. The black area corresponds to a probable natural hazard occurring in the next 100 years, while the orange area corresponds to one occurring in the next 5000 years. The yellow star indicates the location where the pictures in (c) and (d) were taken before and after the 2015 avalanche, respectively. Background satellite image credits: Norwegian Polar Institute.

Longyearbyen, with a population of approximately 2600 inhabitants, is the administrative center of the Svalbard Archipelago and its largest settlement (Figure 2). Formerly a mining town, Longyearbyen's economy mainly relies on tourism, research, and education. Its population is particularly transient, with 43% of residents leaving after the first two years and only 36% staying for more than five years (Statistics Norway, 2022). With 36% of residents being non-Norwegian and over 53 nationalities represented, it is also very international (Statistics Norway, 2022). Climate change in Svalbard is causing rising temperatures, a shift from snowfall to liquid precipitation, the deepening of the permafrost active layer (Osuch et al., 2019), and increased mass movements such as landslides, avalanches, and slope instabilities as well as glacial floods (Dudek et al., 2022; Wieczorek et al., 2023). The location of the settlement, originally chosen because of coal deposits, is now considered risky due to its surrounding steep slopes and its position at the level of the glacial stream fed by two small melting glaciers (Figure 4(a)), which complicate urban development.

### 5.2. The 2015 avalanche: a turning point

On the morning of 19 December 2015, in the midst of the polar night, a significant avalanche descended from the Sukkertoppen mountain onto the Lia neighborhood in Longyearbyen, resulting in the tragic loss of a child and a man (Figure 4, dotted orange patch). This event was unprecedented, as no previous avalanches from Sukkertoppen, or near the town, had caused significant damage to infrastructure or people (Jaedicke et al., 2016). The incident traumatized the community, instilling a sense of vulnerability and changing the perception of the surrounding environment (Léonard et al., 2021). It became a symbol of the adverse effects of climate change and shifted the focus from mitigation towards adaptation to natural hazards, making safety in Longyearbyen a top political priority (Meyer, 2022). Thus, the avalanche served as a ‘focusing event’ (Amundsen et al., 2010; Birkland, 1998) which galvanized public attention and resulted in significant policy changes, many scientific studies, and risk mitigation measures (Birkland, 1998; Longyearbyen Localstyre, 2023).

**Figure 5.**
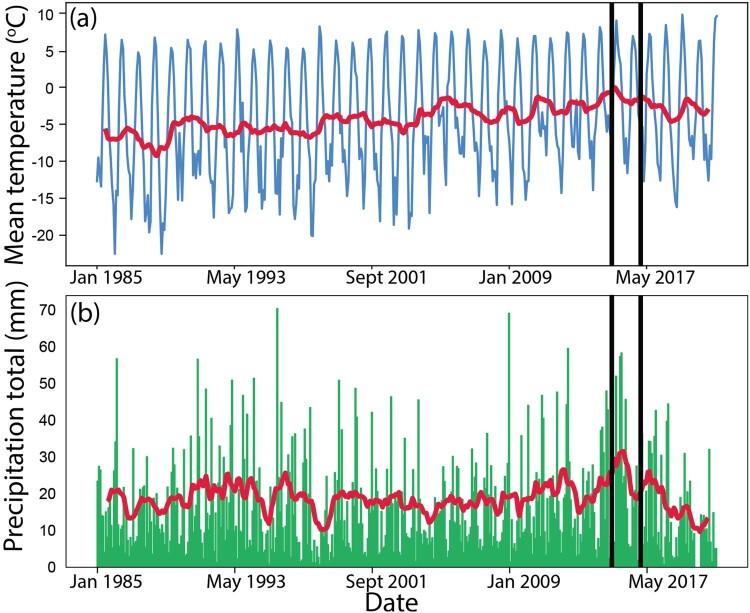
Evolution of climatic conditions in Longyearbyen. (a) Mean surface air temperature and (b) total precipitation in Longyearbyen from January 1985 to August 2022 using monthly data from the Norwegian Meteorological Institute (see Appendix for more detail). The red line in each panel shows the 12-month running mean. Black vertical lines show the moment of the avalanches in December 2015 and February 2017. Data are measured at the Svalbard Airport, located approximately 5 km west of the city center.

Boundary delineation is crucial in risk assessment (Mikes, 2011). However, climatic conditions in Longyearbyen have evolved more rapidly than the understanding of the extent of the danger zone (Hestnes et al., 2016). The available meteorological data indicates a steady increase in air temperatures with clear seasonal variations (Figure 5(a)). In 2015, an intense winter storm characterized by strong winds and snow accumulation patterns (Figure 5(b)) triggered the avalanche (Jaedicke et al., 2016). The impact zone of the avalanche extended 25–60 meters beyond the previously assessed hazard zones (Figure 4(b), black lines), creating an urgent need to reassess risk boundaries. To achieve this, the municipality enlisted the assistance of local and international experts to undertake a comprehensive analysis of avalanche risk. This involved the mapping of new hazard zones (Figure 4(b), orange and black shaded areas), estimation of the likelihood of future avalanches, and an assessment of the vulnerability of the population and critical infrastructure (Longyearbyen Localstyre, 2023; Sydnes et al., 2021).

### 5.3. The 2017 avalanche: a new normal?

In February 2017, a second avalanche destroyed another apartment building (Figure 4, dashed red patch). The failure of monitoring systems installed after the 2015 avalanche to anticipate this event exacerbated the shock of the disaster (Engeset et al., 2020; Sydnes et al., 2021). This was a stark reminder that the risk of natural hazards in Longyearbyen is accelerating and underscored the importance of periodic reassessments of natural hazard risks in rapidly changing environments to ensure that mitigation and adaptation strategies remain effective over time. As an employee of the technical department of the Longyearbyen Community Council remarked in an interview on 4 December 2019: ‘Climate change has been there and changed the premises [for planning]’.

### 5.4. The multi-scalar response

Following these two unprecedented events, the local government adopted in 2018 a new urban planning strategy aimed at protecting the town against future avalanches. This plan included the construction of avalanche barriers on the slopes of Sukkertoppen, a wall at the mountain's base, and the demolition and relocation of 139 housing units in high-risk areas as well as an extension of the ban on building new houses in these neighborhoods. These measures represent a better prepared approach to risk management in Longyearbyen, reflecting a growing recognition of the need to adapt to changing environmental conditions. A year later, this plan had to be revised once again following a major climate report from the Norwegian Climate Service Centre that predicted greater than expected thawing of permafrost, which could pose a threat to the avalanche defenses (Adakudlu et al., 2019). As a result, the foundations of the avalanche fences had to be drilled deeper into the ground. The uncertainty related to the impacts of climate change pose a significant challenge for urban planning, according to experts and the local administration that developed the risk assessments and mitigation measures for the town. As an employee of the technical department of the Longyearbyen Community Council stated: ‘Only in the time I have been living here [2 years], we have moved the risk zones twice. That means what we can build and where has already been changed twice’ (Interview, 4 December 2019).

One of the challenges in defining hazard zones has been the limited understanding of risks involved (Walker et al., 2003), as was highlighted by the 2015 and 2017 events. Early approaches were primarily based on historical events and limited meteorological data such as precipitation and wind (Hestnes, 1994). However, studies have shown that precipitation alone is likely not a sufficient predictor of avalanche occurrence and that avalanche propagation times are longer in colder environments such as Longyearbyen (Hestnes et al., 2016). Prior to 2015, calls were made for increased instrumentation and observation, development of knowledge databases, process-based research, and localization of studies. However, many risk areas continued to be implemented based on past events (Eckerstorfer et al., 2013). Since then, digital terrain models have advanced our understanding of slope and risk conditions, and efforts were underway in 2017 to strengthen predictive modeling capabilities (Bazilchuk, 2017; Norwegian Geotechnical Institute, 2017). Furthermore, although the perception of hazard areas continues to evolve, a lack of dedicated financial resources has left the area exposed, resulting in a mismatch between perceived risks and safety measures (Hestnes et al., 2016). As the climate continues to change in the coming decades (Intergovernmental Science-Policy Platform on Biodiversity & Ecosystem Services, 2019), the static boundaries of the avalanche hazard map may struggle to keep up with the dynamic changes affecting Longyearbyen's climate and society.

### 5.5. Hybridization of urban risks

With changing climate and built environments, hazards other than avalanches are also emerging, such as flooding and erosion caused by the Longyearelva River (Figure 4(a)). Over time, the river has been consolidated into a single wider stream to allow for urban development (Løvaas, 2021). This consolidation, together with increased water flow from glaciers, has led to higher levels of erosion and increased flood potential, posing significant risks to Longyearbyen's infrastructures (Ottem, 2022). Compounding these risks is the fact that the new avalanche risk map sees residential buildings increasingly concentrated around the river.

Another challenge affecting Longyearbyen is the extremely high turnover of its population (only 36% staying for more than five years; Statistics Norway, 2022), which leads to a short collective memory and diffuse perception of risk. It has been shown, from case studies in Iceland, that populations exposed to avalanches may suffer from long term psychological impacts (Thordardottir et al., 2015), which could modify their perception of risk and therefore influence their behavior. However, in Longyearbyen, only a fraction of the current population has experienced the events in 2015 and 2017. As a result, while avalanches have been a topic of discussion in the immediate aftermath of the events, the salience and urgency of these events have diminished over time, making it more challenging to maintain an accurate perception of risk. The case of Longyearbyen underscores the importance of developing scientific inquiries in accordance with changing environment and local knowledge and ensuring continuity in managing risks despite the high turnover of the local decision-makers.

## 6. Summary and perspectives

Through the case of the Arctic region, we challenged the notion of borders as static and omnipotent entities. Traditional approaches to borders emphasize territorial sovereignty, fixed boundaries, and control over resources and movement. Yet, multifaceted contemporary transformations (e.g. melting ice, shifting ecosystems, emerging navigational routes) are occurring at speeds that challenge traditional political and institutional responses (Figure 1). This situation calls for moving beyond state-centric and territory-focused boundary definitions to incorporate ecological dynamics, different knowledge systems, global environmental governance perspectives, and social adaptability to gain practical knowledge (Beck, 2011; Kuus, 2020). To address such a challenge, we used a wide variety of disciplinary perspectives, from natural science to international law and social inquiries. Based on our investigation, we propose here an analysis of the convolution of global, regional and local scales through the lens of boundary-making processes. Finally, we discuss how our exploration of the Arctic and its changing boundaries pave the way for a more resilient Arctic.

### 6.1. Global, regional, and local boundaries in the Arctic

The growing human presence in the Arctic over the past century (Cao et al., 2022) has resulted in the need for drawing boundaries, often leading these emerging human-made boundaries to be subject to disputes within evolving legal and social frameworks. The processes of boundary-making highlighted in this paper illustrate how scientific research often informs policy-making processes, and how political interests shape scientific knowledge production in a loop that becomes challenging to disentangle.

On a global scale, where boundaries are influenced by geopolitical issues, we explored the first internationally shared definition of the Arctic that resulted from the need to define an operational Arctic for policy purposes (Section 3). AMAP created a boundary compromise among various definitions, mainly resulting from a science-informed boundary-making process (Section 3.1). Indeed, AMAP's boundary incorporates elements from geographical boundaries, climatic boundaries, vegetation boundaries, permafrost limits, and major oceanographic features as well as political boundaries. For instance, the boundary follows the arbitrary 60°N parallel over most of Canada, a configuration supported for policy/economic reasons rather than informed upon scientific arguments (Figure 1). This case highlights the interplay of political interests and scientific knowledge production, creating a challenging entanglement. The definition of the Arctic by AMAP not only facilitates effective scientific assessments but also acts as a performative behavior by elevating the region as a distinct entity on the policy agenda and beyond its boundaries (Reiersen et al., 2024; Rødven & Wilson, 2022).

On a regional/national scale, where boundaries are shaped by national identity and interests, we delved into Norway's sovereign and economic rights over the Svalbard maritime zone (Section 4). These rights are dictated by the Svalbard Treaty (1925) and the United Nation Convention on the Law of the Sea (1982). Yet, differences between these two legal frameworks have led to a judicial dispute of access rights to the Svalbard maritime zone. The current approach to settle this dispute lies on the scientific nature of the seabed connection between Norway's mainland and Svalbard (Section 4.1). This approach highlights the need for scientific knowledge in reinforcing, if not legitimizing, policy-based boundaries. Numerous other examples across the Arctic illustrate areas delineated based on scientific knowledge for resource and transit management (Dodds & Hemmings, 2018) or territorial claims based upon geological arguments (Doel et al., 2014). Regarding Svalbard, the Commission on the Limits of the Continental Shelf (CLCS) declared in 2009 that the continental shelf around Svalbard was indeed contiguous to that of the Norwegian mainland (Jensen, 2010). Such a declaration was supported by international collaborative efforts through data collection and analysis. However, per its mandate, the CLCS did not address the Svalbard Treaty applicability (Jensen, 2020) and it was only after the ruling of the Norwegian Supreme Court (Norwegian Supreme Court, 2023) that Norway's economic rights in the region were established. The interpretation and performative practice of sovereignty in such cases depend on power dynamics and narratives, emphasizing the importance of considering these complexities beyond a purely legal framework.

On a local scale, where boundaries are influenced by everyday life experiences, we examined how a series of avalanches have altered risk boundaries and the social fabric of the town of Longyearbyen (Section 5). Following two unprecedented avalanches events, both physical (e.g. construction areas, town's limits) and perceived (e.g. fear of avalanches) have been questioned. In this case, the high population turnover rate reduces residents' collective memory and alters risk perceptions, creating a gap between lived experiences and officially mapped risk boundaries. This portrays the latter as institutionalized knowledge detached from residents' daily practices, highlighting the fluid and constantly reconstructed nature of social realities. The significance of the investigation conducted in section 5 lies in its potential to inform risk management strategies in the polar regions and globally (Ford et al., 2006; Koziell & Gyamtsho, 2023).

### 6.2. Boundaries in a changing world

Today's Arctic experiences multi-scales and trans-boundary changes, from urban development and resource management to geopolitical interests and human-induced climate change. These changes lead to significant environmental disruptions and result in habitat loss for local human and non-human communities (Brown et al., 2023) as well as threat to regions beyond its own boundaries (Hong et al., 2023; Khan et al., 2022; Miner et al., 2022). These changes also cause shifts of the previously defined social, political, and environmental boundaries (Section 3, 4, 5) and put their relevance and suitability into question (Stephenson, 2018). The scientific criteria used to define the bio-physical borders of the Arctic now shape a shrunken region compared to when they were first implemented decades ago (Figure 1). While these shrinking boundaries do not discredit or cast doubt on the scientific assessments that came out of AMAP and other international agreements, they do raise concerns about the relevance of these science-based boundaries in defining a region that matters on the policy agenda (Rødven & Wilson, 2022). Our regional case illustrates this new paradigm by highlighting the international implications of national and domestic politics within the context of changing biophysical boundaries. Indeed, the reduction in summer sea ice extent and Atlantification of the Arctic waters (Figure 3) have resulted in a northward shift of commercially important fish stocks, leading to increased fishing activities and economic interests in the Svalbard maritime zone. As a result, disputes over maritime resources management have intensified, such as the recent disagreement on cod between Norway and the European Union (Ylvisåker, 2021). To maintain control over the resources of this area, Norway's legal sovereignty alone is not enough and requires constant validation through practical (i.e. performative) and functional sovereignty (Section 4). The interconnection between inherently rigid legal boundaries and their practical (and necessary) evolution is further illustrated in the case of Longyearbyen and the way the town has been forced to adapt to changing risks (Section 5). Science-based boundaries (e.g. snow conditions, slopes) and policy-based boundaries (e.g. construction areas) in Longyearbyen have been questioned and redrawn due to changing perceptions, showcasing how social realities contribute to shaping physical boundaries. These dynamics underscore the importance of multifaceted and periodic reassessments of natural hazard risks to ensure that mitigation and adaptation strategies remain effective. The boundary shift phenomenon observed in Longyearbyen is not isolated but rather part of a broader trend affecting other regions of the Arctic and beyond (Ford et al., 2006; van Gevelt et al., 2019). For instance, the decline of sea ice around Greenland has been proposed to favor extreme precipitation events in Svalbard as more moist air gets transported to the west coast of Svalbard (M. Müller et al., 2022). Similarly, the decline of sea ice in the Chukchi (Arctic Ocean between Kamchatka and Alaska) has been shown to favor heatwaves in the Northern Pacific (Carvalho et al., 2021). Thus, risk mapping connects local experiences and knowledge production with regional and global factors, extending beyond geopolitical, environmental, and cartographic dimensions of the Arctic.

In response to rapid human and non-human changes, the evolving boundaries of the Arctic emphasize the relationship between power and knowledge in shaping our reality, highlighting the performative dimensions of discursive practices. These responses suggest that drawing omnipotent boundaries based on a purified knowledge production might be over-simplistic if not idealistic (Strassheim & Kettunen, 2014). Instead, boundaries require reproduction and maintenance, cognitive and symbolic representation, and to be established and performed at the level of lived experiences (Moustard et al., 2021; Poortinga et al., 2019).

### 6.3. Perspectives and recommendations

Addressing climate change and avoiding crossing irreversible limits of human adaptation is a critical challenge of modern society (Rockström et al., 2023). In this paper, we demonstrated the complexity of drawing and maintaining traditional boundaries in the rapidly changing Arctic. We emphasized how climate change is redefining borders and call for moving beyond static definitions and highlighted the dynamic interplay between environmental shifts and daily political decisions (Stephenson, 2018). Building on our investigation of the meaning(s) of the Arctic and its changing boundaries, we propose here three main contributions to guide the discourse on Arctic resilience and adaptive strategies.

**First**, we underline the need to establish a new middle ground for knowledge production in response to the current and potentially irreversible bio-physical transformations. This imperative arises from the necessity to surpass the mere description of change into prescriptions of action. To achieve this, we call for a comprehensive rethinking and redefining of boundaries. Our perspective extends beyond political constructs to encompass human and non-human lives along with the intricate phenomena shaping experiences within the vast terrains of the Arctic. This approach advocates for a re-conceptualization of Arctic geographies, identities, and their interlinks to align with the conditions of the Anthropocene.

**Second**, we emphasize the need to develop a framework capable of comprehensively capturing Arctic transformations across various scales and dimensions. In doing so, we recognize the urgency for prompt and feasible policy decisions made within realistic temporal frameworks. Importantly, this urgency in policy-making must not overshadow the crucial necessity for an inclusive and critically-engaged perspective that welcomes diverse viewpoints and knowledge systems. We firmly believe that this commitment to epistemic diversity and humility serves as the basis for strengthening resilience and crafting governance structures attuned to the multidimensional and rapidly evolving Arctic realities.

**Finally**, we underscore the imperative to transcend conventional constraints of governance confined by national territories. We advocate for a novel governance model that prioritizes the safeguarding of critical functions within the Earth system (Rockström et al., 2024). Recognizing the interdependence and transboundary nature of the Arctic and global ecological processes, our perspective calls for the establishment of extended governance mechanisms and institutions. These should be robust, inclusive, and adept at addressing the far-reaching consequences of Arctic alterations for the equilibrium of the entire planet.

In weaving these three threads together, this paper proposes a distinctive perspective on Arctic governance in the academic discourse. It calls for a re-imagined approach that is both contextually relevant and responsive to the distinctive challenges arising from the transformation of the Arctic and its boundaries in the Anthropocene. These processes are pivotal in shaping the current narrative of international relations, environmental politics, and security concerns in the Arctic and beyond. Embracing diverse knowledge and analytical perspectives is not just a recommendation but a necessity for devising effective strategies to navigate the complexities of the human-induced climate change and its global and pervasive impact on our livelihoods. This is why we advocate for an interdisciplinary and multi-scalar strategy to create environmentally conscious, socially just, and politically feasible policies in light of the Anthropocene's new biophysical reality.

## Notes

1 Defined as the creative and symbolic dimension of the social world, the dimension through which human beings create their ways of living together and their ways of representing their collective life (Thompson, 1982).

2 Article 77: ‘Sedentary species are […] organisms which, at the harvestable stage, either are immobile on or under the sea-bed or are unable to move except in constant physical contact with the sea-bed or the subsoil’.

3 The species with the greatest potential due to their life history characteristics are the beaked rockfish (*Sebastes mentella*), Greenland shark (*Somniosus microcephalus*), Arctic skate (*Amblyraja hyperborea*), Arctic cod (*Boreogadus saida*), Bering flounder (*Hippoglossoides robustus*), Greenland halibut (*Reinhardtius hippoglossoides*), and snow crab (*Chionoectes opilio*; Hollowed et al., 2013).

## Apppendix. Data

To illustrate the change of natural boundaries with time, we use climate simulations from the Community Earth System Model Version 2 (CESM2; Danabasoglu et al., 2020). Specifically, we use the monthly output from the first 50 ensemble members of the CESM2 Large Ensemble (CESM2-LE; Rodgers et al., 2021) that were run from 1850 to 2014 under historical forcing and from 2015 to 2100 following the medium-to-high SSP3-7.0 scenario (O'Neill et al., 2016). We computed the ensemble mean to highlight the forced response to increasing greenhouse gas emissions. Sea ice extent is defined as the area where sea ice concentration is larger than 15%. The 10°C July isotherm line was computed using the 2-m surface air temperature. The extent of continuous permafrost is determined by the region where the maximum annual active layer (i.e. the top layer of soil that thaws during the summer and freezes again during the fall) thickness is between 0 and 3.5 m (Dobiński, 2020).

Maritime boundaries (territorial sea, contiguous zone, exclusive economic zone, and extended continental shelves) were retrieved from the Flanders Marine Institute (2019((@); ecologically or biologically significant marine areas and marine areas of heightened ecological and cultural significance were retrieved from the Conservation of Arctic Flora and Fauna (2016); the polar cod extent was retrieved from Mecklenburg et al. (2016); ocean currents are taken from the Ocean Blue Project at https://oceanblueproject.org/ocean-currents-map/.

To investigate changes in climatic conditions in Longyearbyen, we used air temperature and precipitation totals provided by the Norwegian Meteorological Institute. The data spans the period of January 1985 to August 2022 inclusively and has a monthly resolution. The data was collected from a weather station at the Svalbard Airport. The data on risk perceptions in Longyearbyen were gathered during the course of ethnographic fieldwork between 2018 and 2022 for a research project on climate change impacts, perceptions, and adaptations. The data include interviews with various stakeholders, including planners at the local government (Longyearbyen lokalstyre) sharing their experiences in dealing with the avalanches in 2015 and 2017. The avalanche and risk areas from 1993 and 2004 were assessed with the old avalanche danger maps and current risk boundaries for Longyearbyen (Hestnes et al., 2016). The base orthophotomap, glaciers extent and rivers were provided by Norwegian Polar Institute. The Arctic Digital Elevation Model (Porter et al., 2018) at 3.5x3x5m resolution was used for hypsometry and slope map.
